# Supplementary material for: All-fiber high-speed image detection enabled by deep learning
Source: Nat Commun. 2022 Mar 17;13:1433. doi: 10.1038/s41467-022-29178-8 (PMC8930987; doi:10.1038/s41467-022-29178-8)
Supplement: Supplementary file 1 — Supplementary Information [file 41467_2022_29178_MOESM1_ESM.pdf]

# All-fiber high-speed image detection enabled by deep learning: Supplementary Material.

**Zhoutian Liu[1], Lele Wang[1], Yuan Meng[1], Tiantian He[1], Sifeng He[1], Yousi Yang[1], Liuyue Wang[1], Jiading Tian [1], Dan Li[1,2], Ping Yan[1,2], Mali Gong[1,2], Qiang Liu[1,2] & Qirong Xiao[1,2*]**

[1] State Key Laboratory of Precision Measurement Technology and Instruments, Department of precision instrument, Tsinghua University, Beijing 100084, China

[2] Key Laboratory of Photonic Control Technology, Ministry of Education, Tsinghua University, Beijing 100084, China

Correspondence: xiaoqirong@mail.tsinghua.edu.cn

## Supplementary Note 1: Pulse Transmission

The shapes of the laser pulses before entering the fiber probe and after emerging from the distal end are shown in Supplementary Figure **1**. We see that the full width at half maximum of the pulses broadens from 26.4 ps to 45.1 ps. The broadening is mainly caused by the small modal dispersion existing in the fiber probe, which may slightly influence the performance of the image restoring. This influence can be relieved by replacing the cladding pump way with the core pump way, which couples the illumination light directly into the core of the fiber probe. Due to that the modal dispersion in the core (NA = 0.2) is much lower than that in the cladding (NA = 0.46), the pulse broadening will be much suppressed.

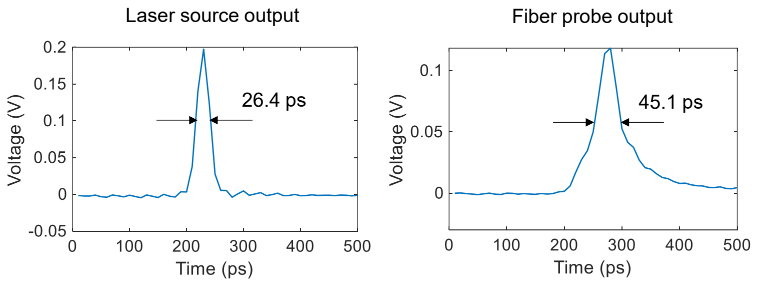


**Supplementary Figure 1.** Pulse shapes detected by the 30 G ultrafast photodetector before the pulses enter the fiber probe and after they emerge from the fiber-end-ball.

The max average power entering the MMF in the experiment is measured to be about 1.5 mW. Considering that the width of the pulses is 26.4 ps, the peak power of the pulses in the fiber is about 3 W. We simulate the evolution of a gaussian pulse with the similar peak power in the 1-km MMF as shown in Supplementary Figure **2**(a) without considering the intermodal dispersion. We see that the broadening caused by the chromatic dispersion and nonlinear effect is negligible. The calculation is based on the non-linear Schrodinger equation (NLSE)[1]. The simulation parameters are as follows. The dispersion coefficient $\beta_2 = 25\times10^{-3}$ ps$^2$m$^{-1}$. The nonlinear coefficient $\gamma = 5.8\times10^{-3}$ W$^{-1}$m$^{-1}$ at 1064 nm in the single mode fiber[2]. Considering $\gamma = n_2\omega/cA_{\mathrm{eff}}$, the $\gamma$ is inversely proportional to the area the fiber core. Thus, in our MMF with a core diameter of 50 µm, the $\gamma$ is about 30 times that of the general single mode fibers with a core diameter of around 9 µm. Therefore, we set the $\gamma = 2\times10^{-4}$ W$^{-1}$m$^{-1}$ in the simulation. Besides, we also calculate the group delays for all the LP modes in the MMF that we used and the results are shown in Supplementary Figure **2**(b). We see that the different modes have different group delays. Compared with the pulse width, the delay differences between different modes are sufficiently large that can cause the energy separating of these modes.

Considering that the fastest and slowest modes have a delay difference of around 50 ns, we can predict that after transmitting through the 1-km MMF, a pulse will eventually split into a pile of isolated sub-pulses over a temporal range of 50 ns due to the intermodal dispersion.

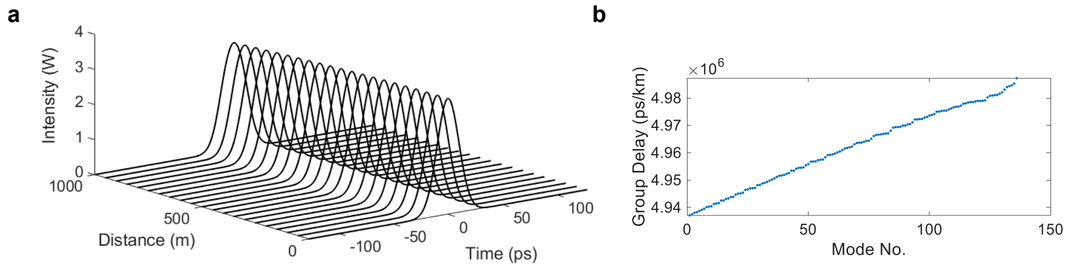


**Supplementary Figure 2.** (a) Evolution of the pulse in the MMF. (b) Group delays of different LP modes calculated by the Finite Difference Method.

## Supplementary Note 2: Fiber Probe

The section refractive index distribution of the fiber probe is shown in Supplementary Figure **3**, which consists of three claddings. The first one is a fluoride doped layer that has a relatively lower index, limiting the signal light in the core. The combination of a second silica cladding and a third low-index coating layer allows the transmission of the illumination light in the second cladding layer.

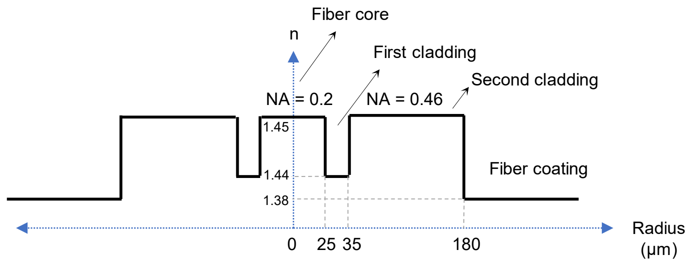


**Supplementary Figure 3.** Section refractive index distribution of the fiber probe.

## Supplementary Note 3: Waveforms

Here we discuss some features of the waveforms. When we put the ten waveforms from Fig. 3 of the main text together in Supplementary Figure **4**, we see that all the sub-pulse peaks are overlapped in time, which proves that the mode dispersion actually dominates in the temporal evolution of the pulses so that the temporal delays of the sub-pulses will only be determined by the group delays of the corresponding modes. We observe from Supplementary Figure **4** that the burst of sub-pulses covers a time range of around 45 ns, a little less than the 50 ns predicted in Supplementary Note 1. This may be attributed to that certain highest-order modes are harder than expected to be excited, or that a trivial deviation of the parameters of the real fiber from the idea values. Besides, we can see that each waveform contains about 40 sub-pulse peaks which is much less than the number of LP modes in the MMF (136, see the calculation in Supplementary Note 1). This may be because that some adjacent modes have very close group delays so that the light energy in these modes is not completely separated in time domain. Instead, they will cause the broadening of each sub-pulse as shown in the figure, which may also contain some information due to the variation of shapes of the sub-pulse.

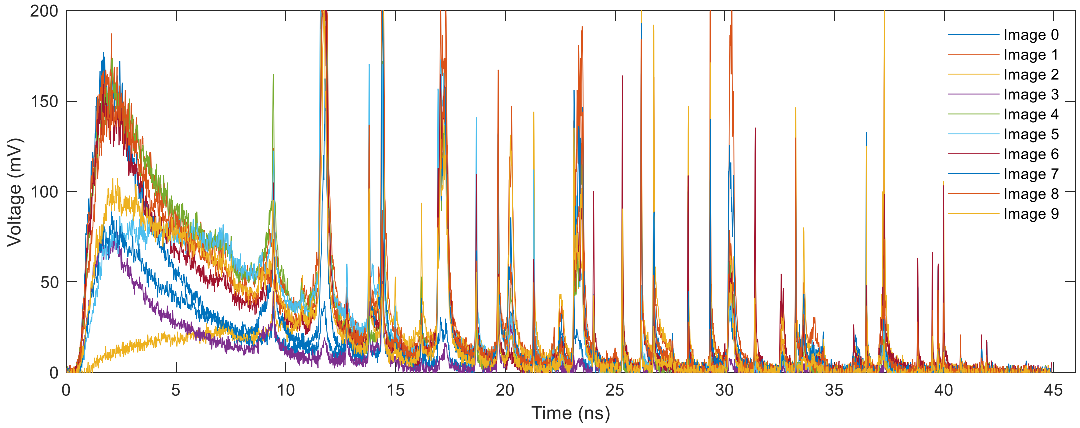


**Supplementary Figure 4.** Superposition of ten waveforms corresponding to different images.

When we reduce the length of the MMF, the waveforms will be shorter due to the reduced modal dispersion as shown in Supplementary Figure **5**. The performance of the system when different lengths are adopted was tested and some recovered results are shown in Supplementary Figure **6**. We see that the images can be restored with high quality until the length is reduced to 400 m, which corresponds to the pulse spreading of approximately 18.7 ns.

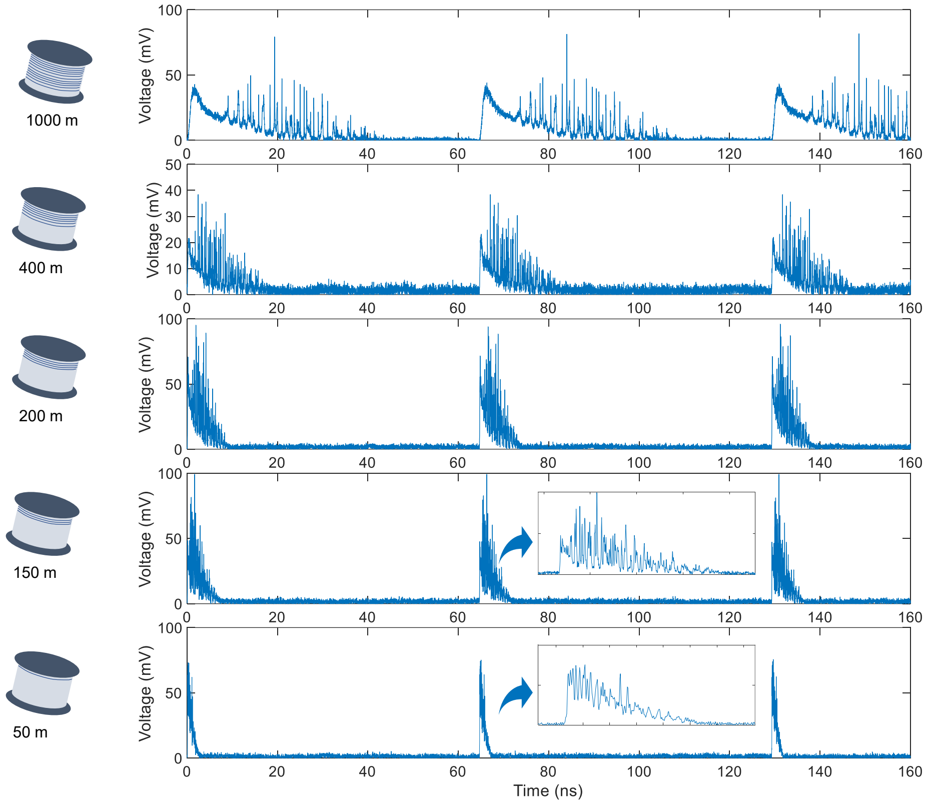


**Supplementary Figure 5.** Detected time signals when different MMF lengths are used.

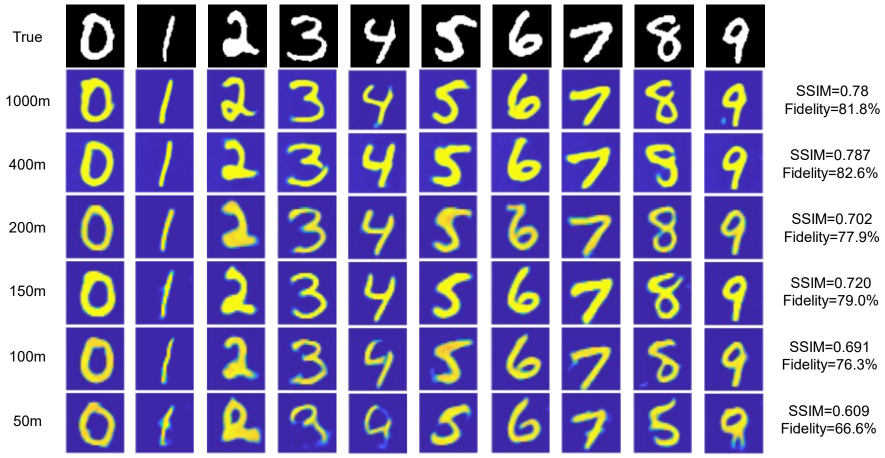


**Supplementary Figure 6.** Some recovering images when different lengths of MMF are used in the system.

## Supplementary Note 4: Imaging of Random objects

In the imaging of random kinds of objects, we used the images that are automatically generated based on the Omniglot dataset to train the system. The 20000 training images are all generated from the transformation of only 1500 original Omniglot images as shown in Supplementary Figure 7a. The transformation includes combinations of three basic operations: patterns moving, rotating and scaling. After testing, we found that the U-Net model is not suitable for recovering images of different types as shown in Supplementary Figure 7b.

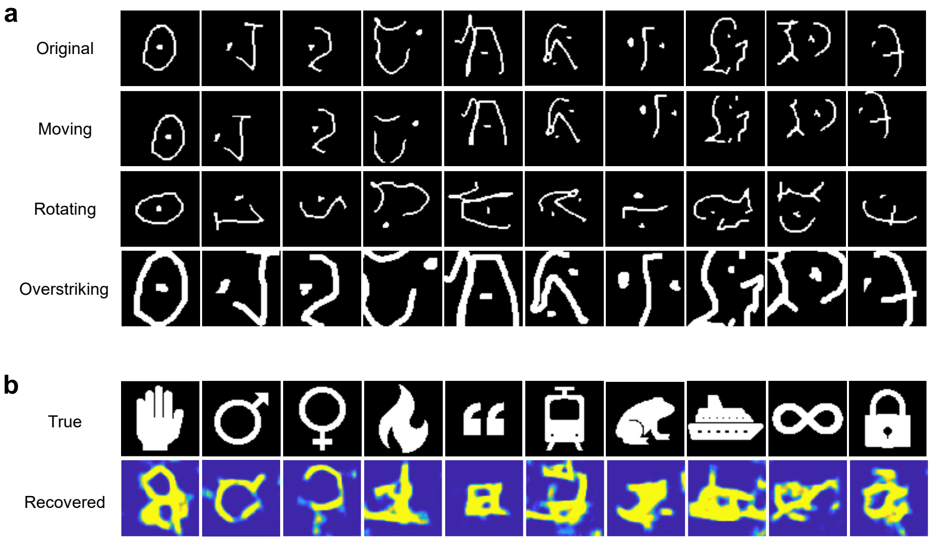


**Supplementary Figure 7.** (a) Some original images from the Omniglot dataset and the generated new images based on the original ones. (b) Some recovered images obtained when using the U-Net model.

The architecture of the Fully-Connected network is simple and shown in Supplementary Figure **8**.

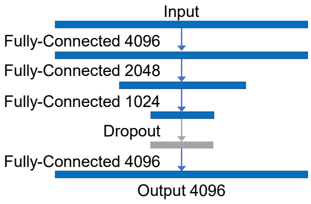


**Supplementary Figure 8.** Fully-Connected structure.

## Supplementary Note 5: Image Classification

We have tried two different network structures for classification of the images of hand-written digits. The first is a CNN network as shown in Supplementary Figure **9**b. The waveforms are reshaped into 64×64 matrices as the input and the categories from 0 – 9 as the output of the CNN. The second is a combination of the U-net (Supplementary Figure **9**a) and the same CNN. The CNN model is pretrained with 60000 different images of hand-written digits so it can act as a digit classifier, which is then used to directly classify the images recovered by the U-net. We use the 20000 image/waveform pairs, including 17000 training data, 2000 valuation data and 1000 testing data to train and test the U-net model. The accuracies corresponding to the two structures: CNN and U-net + CNN, are tested to be 91.5% and 82.0% respectively as shown in Supplementary Figure **10**. The results show that the combination of the two networks provide a higher accuracy, which is consistent with the previous research[3].

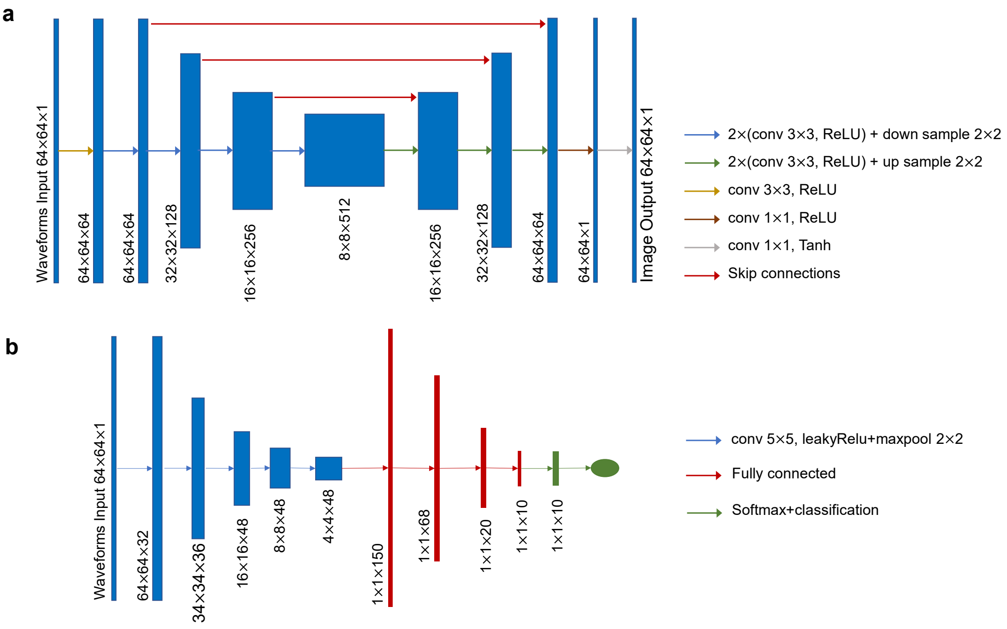


**Supplementary Figure 9.** (a) U-net structure. (b) CNN structure.

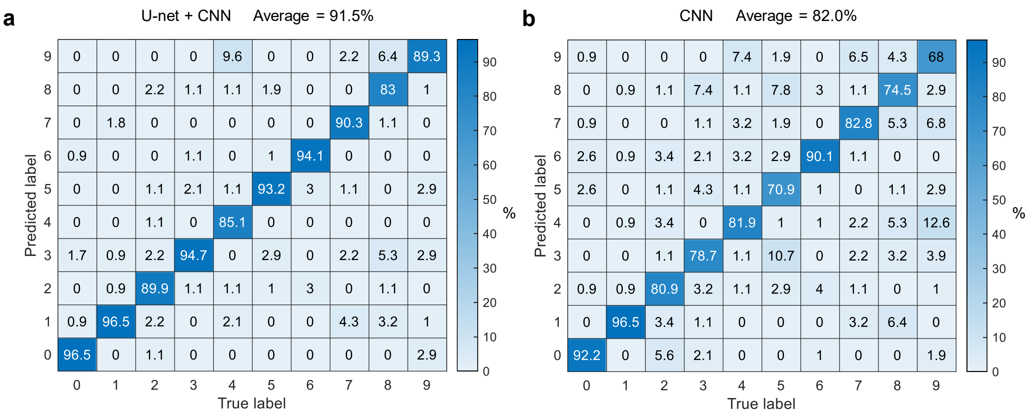


**Supplementary Figure 10.** Confusion matrixes for the classification of 1000 test images using (a) a combination of U-Net and CNN models and (b) the CNN modal alone. These matrixes present the statistics of what proportion of each digit is correctly classified. Each matrix element gives the possibility of instances in which one number (true label) is recognized as another number (predicted label).

## Supplementary Note 6: System Robustness

For the temperature effect, we first collected 10000 image/waveform pairs with the indoor temperature fixed at approximately 25 °C. These sample data were then used to train the neural network. Here, we still used the digit images for training and testing. Next, we changed the environmental temperature from 23 °C to 27 °C by adjusting the air conditioners and the waveforms of new images were collected at different temperatures. We collected 500 sample data every 0.5 °C. In total, 4000 image/waveform pairs were collected and used to test the neural network. The recovery performance at different temperatures is shown by the blue line in Supplementary Figure **11**a. In addition, we verified that this sensitivity can be inhibited by joint training. Joint training involves using the data collected at different temperatures instead of at a fixed temperature to train the neural network. In this way, the trained model can learn to adapt to imaging at different temperatures. To demonstrate this, we again collected 10000 training data with the temperature fixed at 25 °C. Then, we adjusted the temperature from 23 °C to 27 °C continuously and collected another 8000 waveforms of new images at different temperatures, with 1000 data points every 0.5 °C change. Among these 8000 data, 4000 were used as for training and the other 4000 for test. Thus, a total of 14000 data were used to co-train the neural network. Next, the trained model was used to recover the 4000 test images from their waveforms. The results are shown by the red line in Supplementary Figure **11**a. We see that the imaging performance when the temperature changes is largely improved. The average fidelity remains above 70% in a large range of 23.5 °C - 26.5 °C, which is expected to expand further as more sample data collected at different temperatures are used to co-train the neural network. Some restored images are shown in Supplementary Figure **12** at different temperatures using the joint training. We see that the images can be restored fairly well from $T$ = 23.5 °C to $T$ = 26.5 °C. Considering that the general indoor working temperature is within this range, our system owns certain practicability. In practical applications, the 1-km MMF and the laser source can be packed in a thermostatic container, which can further improve the robustness to the environmental temperature.

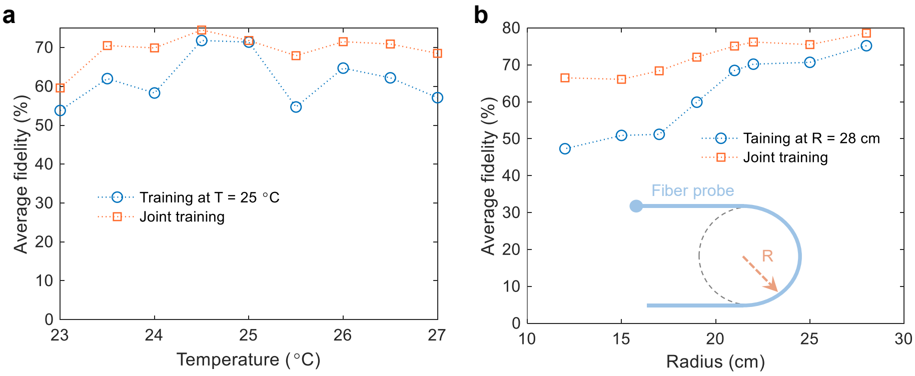


**Supplementary Figure 11.** (a) Imaging performance at different temperatures. The blue line shows the results when the system was calibrated at a fixed temperature. The red line shows the results when the joint training was used. (b) Imaging performance at different bending radii. The blue line shows the results when the system was calibrated at a fixed bending state. The red line shows the results when joint training was used. The inset is the sketch of the bending state of the fiber probe.

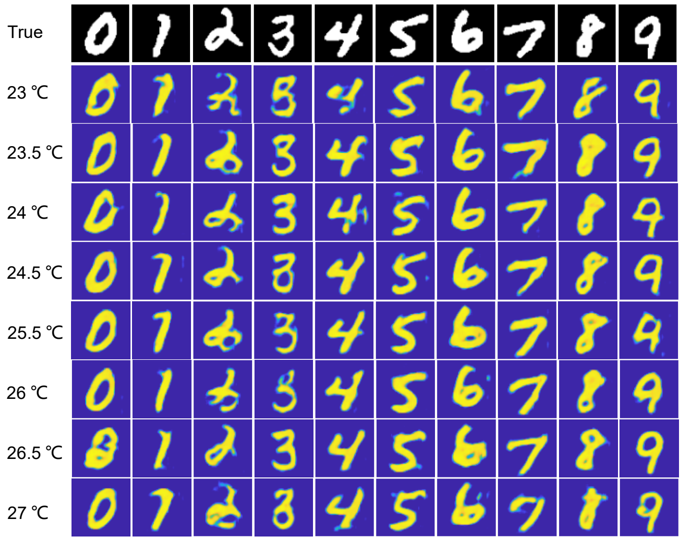


**Supplementary Figure 12.** Several recovered images at different temperatures after joint training.

To investigate the bending effect, we fixed the fiber-end-ball and bent the fiber probe into a semicircle as shown in the inset of Supplementary Figure **11**b. We use the bending radius $R$ to measure the bending degree. Similarly, 10000 image/waveform pairs are used to calibrate the system when the bending radius is fixed at 28 cm. Then, the fiber was bent and the radius was changed from 28 cm to 12 cm and we collected test data under 8 different bending states, with 500 data per state. These data were used for testing. The quality of the restored images at different bending states is shown in Supplementary Figure **11**b by the blue line. We demonstrate the joint training in the same way: changing the bending radius from 28 cm to 12 cm and meanwhile collecting 8000 data at different bending states: 4000 for training and the other 4000 for testing. The initial 10000 data collected under $R = 28$ cm and the 4000 training data were combined together to co-train the network. Then it was used to recover the other 4000 images. The results are shown as the red line in Supplementary Figure **11**b. We see that the influence of bending is much weakened. Some restored images at different states are shown in Supplementary Figure **13**. We see that the images can be mostly restored until $R = 19$ cm.

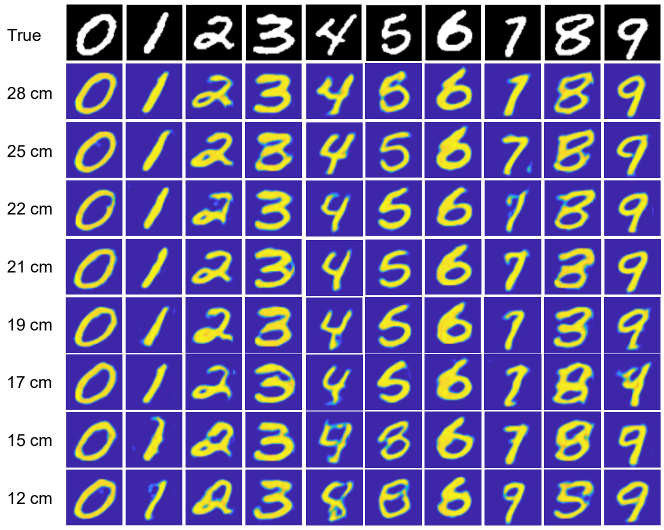


**Supplementary Figure 13.** Several recovered images at different bending states after joint training.

## Supplementary Note 7: Individual Illumination

Like most of the previous researches, we have tried to use an individual source to illuminate the images and an objective to couple light into the fiber probe as shown in Supplementary Figure **14**. The source is collimated by a lens and illuminates the DMD where the images with a size of around 5×5 mm$^2$ are displayed. The reflected light is coupled into the 1-km MMF via a 40× objective. The rest parts of the system are totally same with that presented in the main text. Compared with the all-fiber system, this individual-illumination system has the advantages of providing a brighter illumination and is suitable for detecting relatively large objects due to the use of an objective. After the same training and testing, the recovery results show a fidelity of 83.0% and SSIM of 0.76. The comparison of the recovery performance of the two different systems are shown in Supplementary Figure **15**(a), which indicates that the two different illumination methods have similar performance and proves the high adaptability of our proposed method. The recovery of some other types of images of this system is also tested and shown in Supplementary Figure **15**(b). Besides, we also test its classification performance and the confuse matrices are shown in Supplementary Figure **16**.

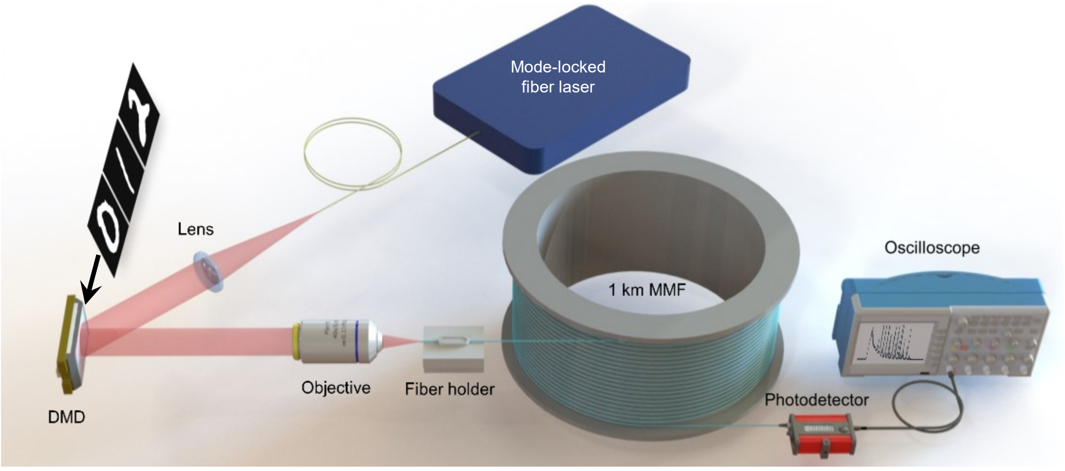


**Supplementary Figure 14.** Schematic of the experiment setup with an individual illumination.

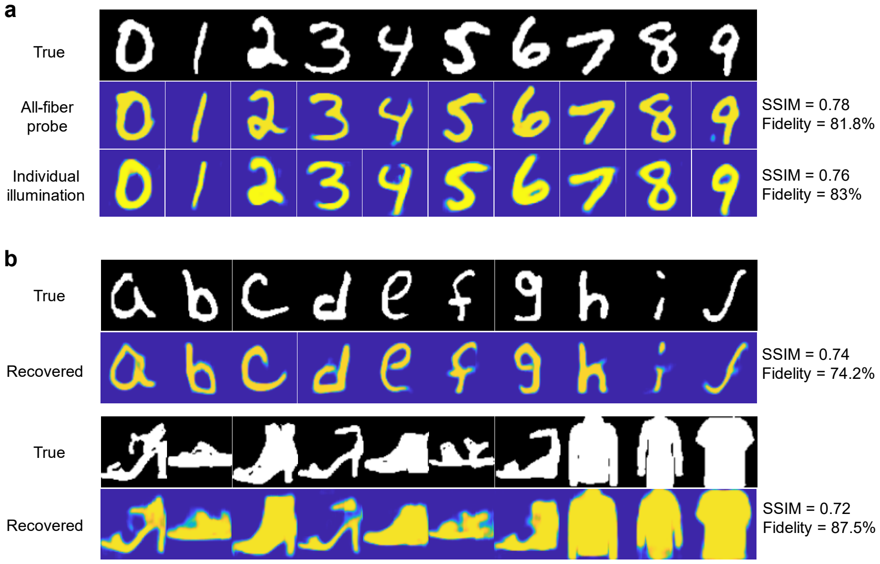


**Supplementary Figure 15.** (a) Comparison of recovery performance between the all-system and the system with an individual illumination. (b) Some example images of letters and clothes and the corrosponding recovered results.

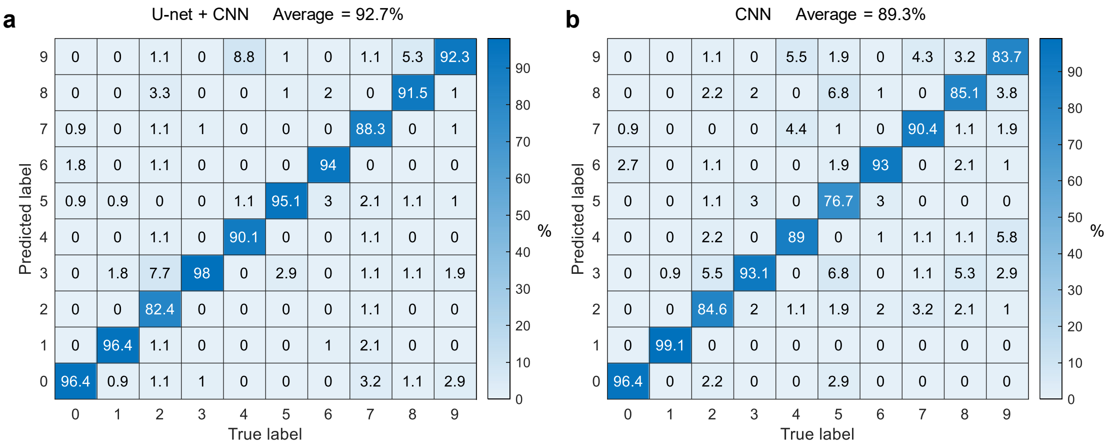


**Supplementary Figure 16.** Confusion matrixes for the system that uses the individual illumination setup. (a) The result of using the combination of U-net and CNN networks. (b) The result of using simply the CNN network. The average accuracy for 1000 test images of digits are shown in the top.

## Supplementary Note 8: System Fabrication

The fabrication procedure includes 4 steps as shown below.

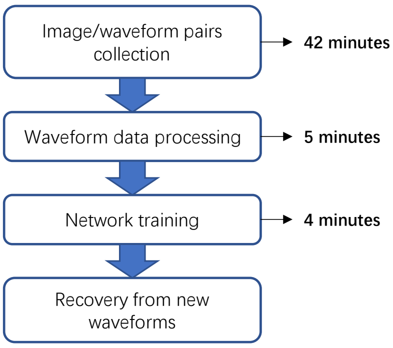


**Supplementary Figure 17.** Calibration and testing procedures and the required time for each step.

The first step is collecting waveform/image pairs for training. To explore the minimum number of pairs that is required for recovery, we trained the network with different number of samples and the results are in Supplementary Figure **18**a. We see that 10000 samples are adequate for acquiring the optimal performance. Thus, at least 10000 waveform/image pairs should be collected. The training process with 10000 samples is shown in Supplementary Figure **18**b. The model restrained after approximately 15 iterations, which took 4 minutes.

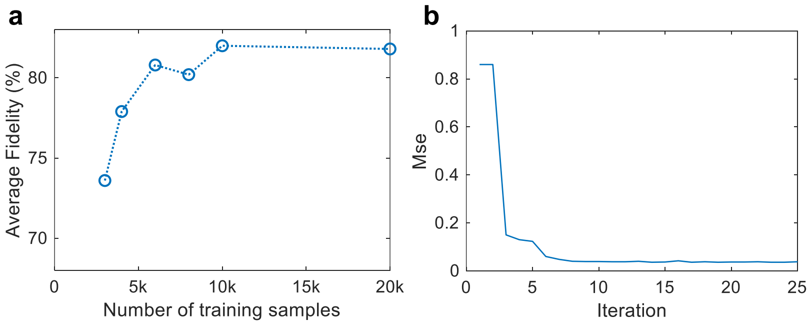


**Supplementary Figure 18.** (a) Performance when the network is trained with different number of samples. (b) Training process.

## Supplementary Note 9: Grey-scale Image Detection

Obviously, the waveforms can carry partial information of the gray scale of the objects. However, the DMD can only display binary images in an ultra-short time duration: the micromirror of the DMD relies on rapid vibration to produce a gray-scale modulation. Thus, we adopted an alternative method that displayed pseudo gray scale images on the DMD: Because the pixel in DMD can only present 1 or 0, in order to display a pseudo gray scale image, we transform the gray scale of an image into the dense of the pixel 1. In detail, we use a 3×3 pixel block on the DMD to represent one pixel of the gray-scale images. According to the number of pixel 1 in a block, it can present 9 levels of gray scale (0 - 9) as shown in Supplementary Figure **19**. Due to the resolution of the displayed images (192×192) is far beyond the resolution of our system, a 3×3 pixel block can act as one gray scale pixel where the reflected intensity of light is in direct proportion to the number of pixels 1. Due to the pseudo gray scale images (192×192) require a much larger region on the DMD, and the single fiber end can only detect a small region on it, we use a lens in front of the fiber end to expand the illumination beam as shown in Supplementary Figure **20**. Thus, a large region of the DMD surface can be illuminated and detected. An image with much more pixels can be displayed there.

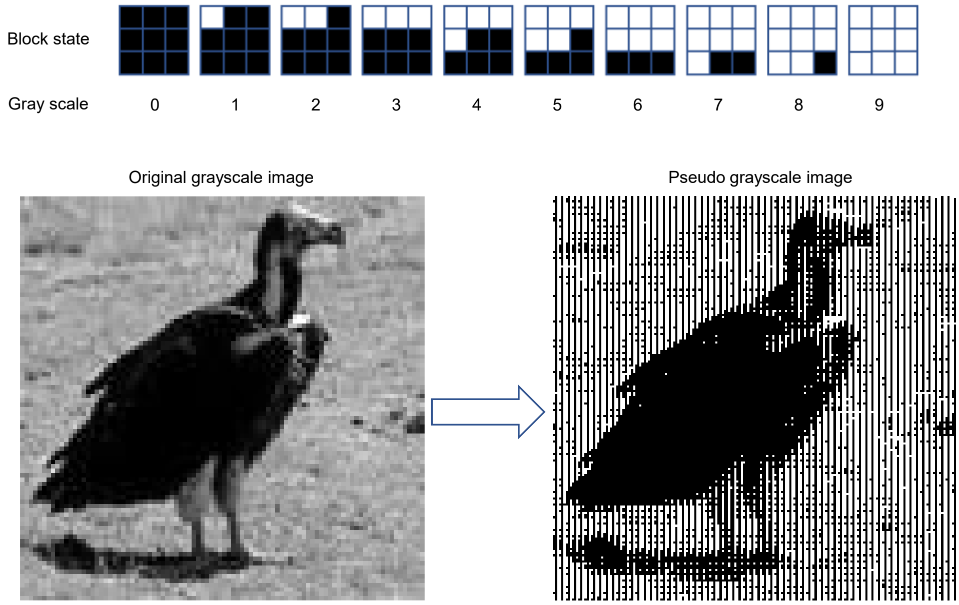


**Supplementary Figure 19.** Explaining of pseudo gray scale images. (Credit: U.S. Fish and Wildlife Service)

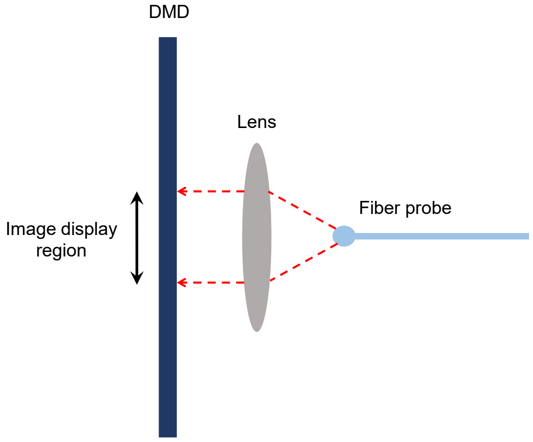


**Supplementary Figure 20.** Function of an external lens.

In this way, we calibrated and tested the system with the gray scale images from the ImageNet database, which consists of images of different natural scenes. 29000 images were for training and another 1000 for testing. We adopted the fully connected network model. Some test results are shown in Supplementary Figure **21**, which proves that the gray scale information can still be partially reconstructed. We believe the use of fibers with more modes can help to reconstruct images with better resolutions.

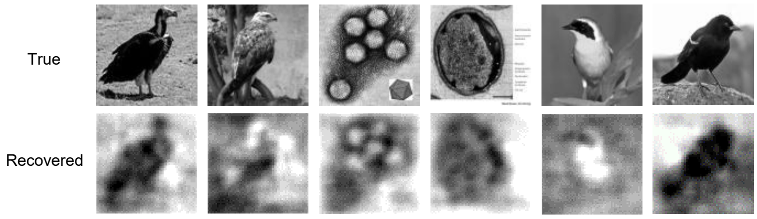


**Supplementary Figure 21.** Some recovered images from the ImageNet database. (Credit: U.S. Fish and Wildlife Service, A.jo, Graham Beards, CC BY-SA 3.0 (https://commons.wikimedia.org/w/index.php?curid=317838))

## Supplementary References

1. Agrawal GP. Nonlinear fiber optics. In: *Nonlinear Science at the Dawn of the 21st Century*). Springer (2000).
2. Kruglov V, Peacock A, Harvey JD, Dudley JM. Self-similar propagation of parabolic pulses in normal-dispersion fiber amplifiers. *JOSA B* **19**, 461-469 (2002).
3. Borhani N, Kakkava E, Moser C, Psaltis D. Learning to see through multimode fibers. *Optica* **5**, 960-966 (2018).
